# ADAPTING TO HEALTH CHANGE: STUDENT RESPONSES TO DIFFERENT DELIVERY MODALITIES

**DOI:** 10.1093/geroni/igae098.3104

**Published:** 2024-12-31

**Authors:** Keith Kleszynski, Lee Jennings, Shirley James

**Affiliations:** University of Oklahoma Health Sciences Center, Oklahoma City, Oklahoma, United States; University of Oklahoma Health Sciences Center, Oklahoma City, Oklahoma, United States; University of Oklahoma Health Sciences Center, Oklahoma City, Oklahoma, United States

## Abstract

**Background:**

Adapting to Health Change (AHC) is a two-hour aging sensitivity simulation training designed to improve student empathy and understanding of common age- and chronic illness-related functional changes. AHC includes role-play with props and 4 activity stations: hearing loss, neuropathy and dexterity, vision loss and medication management, and mobility and transfer. Training is facilitated by interdisciplinary faculty and staff.

**Methods:**

AHC was delivered in-person in 2 sessions to 38 speech pathology students and delivered virtually to 136 social work students in 4 sessions. The simulation was modified for online education by using common household items for props in the activity stations. Post-training evaluation results were analyzed between delivery modalities and student groups.

**Results:**

Students strongly agreed that the simulations helped them better understand how it feels to age with health changes (79%) and potential stereotypes and biases associated with aging (80%). Students strongly agreed the simulations provided useful insight into the aging experience (82%) and that the experience will help them develop into better health care providers of patients adapting to changes associated with aging (83%). The majority of students felt that their knowledge of age-related challenges had improved (94%). There were no significant differences between student groups or between those who received virtual versus in-person delivery.

**Conclusion:**

Aging empathy simulations improve student understanding of age-related health changes and can be effectively delivered in-person or online, making wider delivery to health professions students using virtual training platforms more feasible.

